# Phylogeny and antiquity of M macrohaplogroup inferred from complete mt DNA sequence of Indian specific lineages

**DOI:** 10.1186/1471-2148-6-9

**Published:** 2006-02-07

**Authors:** Revathi Rajkumar, Jheelam Banerjee, Hima Bindu Gunturi, R Trivedi, VK Kashyap

**Affiliations:** 1 National DNA Analysis Centre, Central Forensic Science Laboratory, 30 Gorachand Road, Kolkata- 70014, India

After the publication of this work [1] it was brought to our attention that the GenBank numbers of the 23 M haplogroup sequences had not been provided. The GenBank accession numbers for these sequences are as follows (See Table 1)

**Table 1:** 

**S. No.**	**Sample I.D**	**GenBank accession number**
1	Gow96	DQ246811
2	I.IB306	DQ246812
3	I.B.Ban	DQ246813
4	I.Bho134	DQ246814
5	I.BKur	DQ246815
6	I.BYad	DQ246816
7	I.Chen	DQ246817
8	I.Chr252	DQ246818
9	I.Ho69	DQ246819
10	I.Katk	DQ246820
11	I.Kom4	DQ246821
12	I.Kur126	DQ246822
13	I.Kur150	DQ246823
14	I.Lam8	DQ246824
15	I.Lam18	DQ246825
16	I.Lyn180	DQ246826
17	I.Mah6	DQ246827
18	I.Mus112	DQ246828
19	I.Mus114	DQ246829
20	I.NGond	DQ246830
21	I.Paw50	DQ246831
22	I.Raj90	DQ246832
23	I.Sao	DQ246833

We regret any inconvenience caused to researchers.
